# From commensal to pathogen: recent advances in the pathogenicity and clinical implications of *Streptococcus anginosus*

**DOI:** 10.3389/fmicb.2026.1953186

**Published:** 2026-09-11

**Authors:** Ying Chen, Hongjie Zheng, Wenhua Huang, Shaolong Chen, Yongqiang Jiang, Shengwei Zhang, Peng Liu

**Affiliations:** 1 School of Light Industry Science and Engineering, Beijing Technology and Business University, Beijing, China; 2 State Key Laboratory of Pathogen and Biosecurity, Academy of Military Medical Sciences, Beijing, China; 3 Department of Clinical Laboratory, Dongfang Hospital, Beijing University of Chinese Medicine, Beijing, China

**Keywords:** clinical infection, gastric cancer, pathogenicity, *Streptococcus anginosus*, virulence factors

## Abstract

*Streptococcus anginosus* (*S. anginosus*, SA), a facultatively anaerobic coccus, functions as both a commensal bacterium and an opportunistic pathogen. It is frequently associated with localized suppurative infections and abscess formation. Increasing evidence implicates it as an emerging pathogen that can trigger gastritis and gastric cancer. This review synthesizes current knowledge with emphasis on pathogenic mechanisms of infections and gastric cancer, as well as clinical management. Pathogenic mechanisms include bacterial adhesion, microbe-host interactions, immune microenvironment remodeling, candidate metabolites and extracellular vesicles as potential mediators. We further explore its possible involvement in the oral-gastric axis and niche adaptation, as well as antimicrobial resistance. Current and emerging diagnostic, therapeutic and preventive strategies targeting *S. anginosus* are systematically evaluated. This review highlights the context-dependent pathogenicity of *S. anginosus* and its ecological interactions, providing a critical foundation for the development of robust diagnostic biomarkers and precision therapeutic strategies.

## 1 Introduction

*Streptococcus anginosus* (*S. anginosus*) primarily colonizes the oral cavity, upper respiratory tract, and gastrointestinal tract (Whiley et al., 1992; Xia et al., 2025). Although it was historically regarded as a commensal organism, it is now increasingly recognized as a clinically significant opportunistic pathogen (Asam and Spellerberg, 2014). It has been implicated in a broad spectrum of pyogenic infections, including abscess formation, bacteremia, and infective endocarditis, particularly in immunocompromised individuals and patients with underlying comorbidities (Junckerstorff et al., 2014; Finn et al., 2018). This established infectious disease framework has stimulated considerable interest in its virulence attributes, host-microbe interactions, and niche adaptation across diverse anatomical sites (Asam and Spellerberg, 2014; Kuryłek et al., 2022).

In addition to its classical role in suppurative infection, mounting evidence has implicated *S. anginosus* in gastric pathologies, including superficial gastritis (SG), atrophic gastritis (AG), and gastric cancer (GC) (Qian et al., 2024; Xia et al., 2025). Notably, the available evidence is not limited to correlative observations (Zhou et al., 2022; Yuan et al., 2024). For example, in gastric cancer samples, *S. anginosus* is not only enriched but also contributes to tumor progression by colonizing the gastric mucosa, and promoting the progression of precancerous gastric lesions (Fu et al., 2024). These findings raise the possibility that *S. anginosus* may represent more than a bystander microbial signature, instead acting as a direct promoter of gastric cancer progression under experimental conditions.

Recent studies further suggest that the pathogenic potential of *S. anginosus* may extend beyond intact bacterial cells. In this context, *S. anginosus*-derived extracellular vesicles (SA-EVs) have emerged as functionally active mediators of virulence, a role that is increasingly well-supported by evidence. Gong et al. (2025, 2026) demonstrated that SA-EVs contribute to epithelial injury, amplify inflammatory responses, and dysregulate host immunity. These findings suggest a vesicle-dependent mechanism that links bacterial colonization to mucosal damage. Nevertheless, the roles of SA-EVs in human disease contexts, including gastric cancer progression, remain to be further validated.

Here, we synthesize current knowledge on *S. anginosus*’s role in clinical infections and gastric disease, focusing on its pathogenic mechanisms and clinical management. We explore its colonization along the oral-gastric axis, inflammation induction, tumor promotion, immune modulation, and metabolic crosstalk with the host. Furthermore, we evaluate existing therapeutic strategies targeting *S. anginosus* and propose future directions for research and clinical translation to address the unresolved questions.

## 2 Taxonomy, phylogeny, and biological characteristics of *S. anginosus*

**FIGURE 1:**
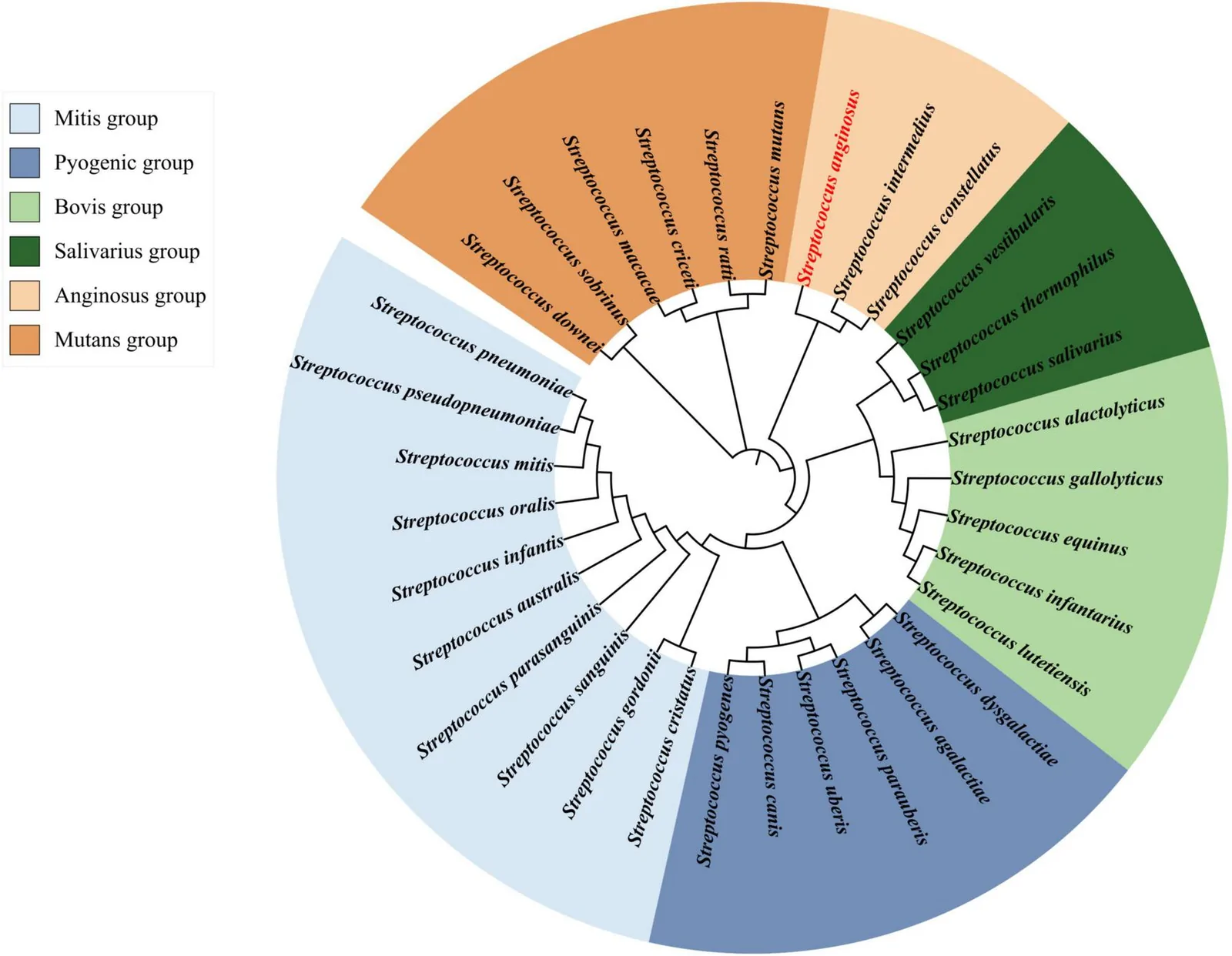
A phylogenetic tree of the genus *Streptococcus* based upon the 16S rRNA gene sequences. The genus *Streptococcus* comprises six major phylogenetic groups and 33 species, with *Streptococcus anginosus* classified within the Anginosus group. Distinct colors indicate different phylogenetic groups, and terminal branches distinguish individual species within each group. Constructed with MEGA 12 and visualized using the Interactive Tree of Life (iTOL) online tool. Taxon sampling employed representative-type taxa per major streptococcal clade, following the established 16S rRNA-based six-group taxonomy. Clinically relevant human-associated species with high-quality public reference sequences were prioritized, while poorly characterized environmental taxa outside the scope of this study were excluded.

*S. anginosus* is a species of the genus *Streptococcus*, comprising six phylogenetic subgroups: Mitis group, Mutans group, Anginosus group, Salivarius group, Bovis group, and Pyogenic group (Figure 1). *S. anginosus* has traditionally been classified within the *S. anginosus* group (SAG), which also includes *Streptococcus constellatus* and *Streptococcus intermedius* (Whiley et al., 1992). Nevertheless, increasing taxonomic and clinical evidence has highlighted its distinct biological identity, as evidenced by unique virulence determinants, specific infection phenotypes, and characteristic antimicrobial patterns (Junckerstorff et al., 2014; Finn et al., 2018; Wu and Zheng, 2020).

Morphologically, *S. anginosus* is a Gram-positive, non-motile, non-spore-forming coccus that typically occurs in pairs or short chains, with a cell diameter ranging from 0.6 to 1.0 μm (Xia et al., 2025). Physiologically, *S. anginosus* is a catalase-negative *Streptococcus* that grows under both aerobic and anaerobic conditions (Ruoff, 1988). This characteristic endows it with considerable environmental adaptability and broad colonization ability. It also exhibits heterogeneous hemolytic phenotypes, including α-, β-, and γ-hemolysis. Moreover, *S. anginosus* is capable of forming biofilms that establish a protective microenvironment, thereby facilitating immune evasion and increasing antibiotic tolerance (Petersen et al., 2006).

At the molecular level, this organism produces an array of virulence factors, including hemolysins, prothrombin activators, and adhesion molecules (Kuryłek et al., 2022), which collectively mediate colonization, host invasion, and immune evasion. In addition, it secretes enzymes such as hyaluronidase that degrade the host extracellular matrix, thereby promoting tissue invasion and infection progression. Furthermore, its metabolic versatility enables the efficient utilization of host-derived substrates and confers a competitive advantage within complex microbial communities. Collectively, these biological traits help explain how *S. anginosus* shifts from a harmless resident to an opportunistic pathogen.

## 3 Core virulence factors of *S. anginosus*

Available evidence indicates that the pathogenicity of *S. anginosus* is multifactorial and mediated by diverse virulence-associated determinants, including adhesins, cytotoxic factors, metabolic products, and extracellular vesicle-associated components. These factors act together, potentially promoting host colonization, tissue injury, inflammatory responses, and disease progression in different anatomical sites (Table 1).

**TABLE 1:** Virulence-associated factors of *S. anginosus*.

Virulence-associated factor	Type	Main roles	Pathogenic propensity	References
FBP62	Adhesin	Mediates fibronectin binding, host adhesion, colonization, and abscess-associated pathogenicity	Classical infection	Kodama et al., 2018
SLS	Cytotoxin	Induces host cell injury, hemolysis, tissue necrosis, and inflammatory responses	Classical infection	Asam et al., 2015; Tabata et al., 2019
H_2_S	Metabolic product	Promotes cytotoxicity, epithelial/tissue injury, and inflammatory microenvironmental alteration	Classical infection	Yoshida et al., 2002, 2003
TMPC	Adhesin	Promotes adhesion to gastric epithelial cells and activates the ANXA2/MAPK signaling pathway	Gastric mucosal injury	Fu et al., 2024
Methionine	Metabolic product	May promote host methionine-cycle flux, SAM synthesis, and tumor-associated metabolic reprogramming	Gastric mucosal injury	Zhou et al., 2026
Succinate	Metabolic product	May activate SUCNR1-associated signaling and participate in immune modulation and tumor microenvironment-related signaling	Gastric mucosal injury	Liao et al., 2026
SA-EVs	Extracellular vesicles	Deliver virulence-associated cargo and mediate epithelial injury, inflammatory amplification, barrier disruption, and immune dysregulation	Gastric mucosal injury	Gong et al., 2026

### 3.1 Adhesion and colonization factors

Adhesion to the host surface is a fundamental step in the pathogenetic process of *S. anginosus*, and several studies have supported the role of specific adhesins in this process. The fibronectin-binding protein, FBP62 of *S. anginosus* has been characterized as a non-canonical adhesin, as it lacks both a canonical N-terminal signal peptide and a C-terminal cell wall-anchoring motif -features typical of classical surface proteins. Kodama et al. (2018) reported that recombinant FBP62 binds fibronectin in a dose-dependent manner and competitively inhibits the binding of *S. anginosus* to fibronectin. In addition, an isogenic Δ*Fbp62* mutant showed reduced adherence to HEp-2 and DOK cells, whereas animal experiments indicated attenuated virulence, with reduced lethality and impaired abscess formation relative to the parental strain. These findings indicate that FBP62 is an important colonization-associated factor that likely contributes to host attachment and abscess-associated pathogenicity of *S. anginosus*. However, its distribution across diverse clinical isolates and its relative contribution in different anatomical niches remain to be clarified.

Another *S. anginosus* surface protein, *T. pallidum* membrane protein C (TMPC), can bind Annexin A2 (ANXA2) receptor on gastric epithelial cells (Fu et al., 2024). Interaction of TMPC with ANXA2 mediated attachment and colonization of *S. anginosus* and induced mitogen-activated protein kinase (MAPK) activation, then induced gastritis and dysplasia in mice. At present, the support for TMPC as an adhesion factor comes only from experimental models involving interaction with gastric epithelial cells. However, it is not clear whether this adhesion molecule has a similar function in non-gastric infection sites. Overall, current evidence indicates that adhesin-mediated colonization is an important early step linking mucosal adhesion to subsequent tissue injury.

### 3.2 Cytotoxic factors

*S. anginosus* possesses virulence traits that may promote the host cells injury and suppurative inflammation. Some *S. anginosus* strains display a prominent β-hemolytic phenotype, and the genes responsible for β-hemolysis are *sag* gene clusters, which encode for a variant of streptolysin S (SLS) (Asam et al., 2013; Tabata et al., 2013). The SLS of *S. anginosus* is a broad-spectrum hemolysin that can lyse erythrocytes from various species (e.g., horse, bovine, rabbit, and chicken) (Asam et al., 2015). Moreover, its hemolytic activity can change with environmental conditions (such as temperature and glucose supply conditions). In β-hemolytic *S. anginosus*, SLS has been detected in the extracellular milieu and has been associated with host cell damage and local tissue injury (Tabata et al., 2019). These observations support a role for SLS in enhancing inflammatory necrosis and suppurative pathology. However, the extent to which SLS contributes across strains and infection contexts remains incompletely defined.

Hydrogen sulfide (H_2_S) production represents another tissue-damaging trait that may contribute to the pyogenic behavior of *S. anginosus*. This organism expresses a *lcd*-encoded β-class C-S lyase that catalyzes H_2_S generation from L-cysteine (Yoshida et al., 2002). Comparative studies have suggested that *S. anginosus* has a relatively high H_2_S-producing capacity among several other oral streptococcal species (Yoshida et al., 2003). As H_2_S can exert cytotoxic effects at high concentrations, including hemolysis-related injury, this metabolic output may plausibly amplify local tissue damage (Beauchamp et al., 1984). Another study also showed that *lcd* transcription was significantly increased in abscess tissue, and that exogenous L-cysteine supplementation enlarged *S. anginosus*-induced abscesses *in vivo* (Takahashi et al., 2011). Nonetheless, the contribution of the ß-class C-S lyase/H_2_S axis to disease severity is still not fully resolved, as it may be influenced by local nutrient conditions and strain background.

### 3.3 Metabolites

Microbe-derived metabolites serve as pivotal biochemical messengers that mediate microbe-host crosstalk, and are increasingly recognized as key drivers of tumorigenesis, as well as promising targets for cancer diagnosis and therapy (Yang et al., 2026). Recent studies suggest that *S. anginosus* may influence host pathological processes through metabolic interactions, with its secreted metabolites methionine and succinate emerging as key candidates that potentially promote gastric cancer (Liao et al., 2026; Zhou et al., 2026). These findings point to the possibility that bacterial metabolic activity could reshape epithelial signaling and the local microenvironment in ways that contribute to disease progression. However, this line of evidence should, at present, be interpreted cautiously. The extent to which these metabolites are uniquely attributable to *S. anginosus in vivo*, their quantitative relevance within the gastric niche, and their causal contribution to human gastric carcinogenesis remain to be established. Therefore, in the context of core virulence traits, these metabolites are better understood as emerging host-interacting factors rather than established oncogenic drivers.

### 3.4 Extracellular vesicles

Extracellular vesicles derived from *S. anginosus* (SA-EVs) are increasingly recognized as important virulence-associated mediators that extend the pathogenic potential of this organism beyond direct bacterial contact. Evidence from gastric disease models suggests that SA-EVs accumulate in gastric tissue and are internalized by epithelial cells. This process induces both acute and chronic inflammatory injury, characterized by neutrophil infiltration, increased production of proinflammatory cytokines, disruption of tight junction proteins, and progressive mucosal damage (Gong et al., 2026). Multi-omics analyses further support a mechanistic role for SA-EVs in disease pathogenesis. Specifically, they revealed an enrichment of virulence-associated proteins TMPC and FBP62. They also showed activation of macrophage polarization- and cytokine receptor-related pathways, as well as metabolic disturbances associated with inflammatory responses. Notably, deletion of either *Tmpc* or *Fbp62* markedly attenuates the pathogenic effects of SA-EVs *in vivo*, as evidenced by reduced gastric inflammation, cytokine production, and macrophage infiltration. Together, these findings establish SA-EVs as key effectors in gastritis and suggest that their vesicular cargo directly contributes to disruption of epithelial barrier, immune activation, and metabolic dysregulation.

Beyond local tissue injury, SA-EVs may also serve as systemic immunomodulatory factors in microbiome-associated diseases such as systemic lupus erythematosus (SLE). In SLE patients, increased abundance of *S. anginosus* is linked to higher disease activity and enhanced natural killer (NK) cell cytotoxicity, further supporting a link between microbial dysbiosis and immune dysfunction. Mechanistic studies indicate that SA-EVs directly stimulate NK cells, promoting granzyme B and TNF-α production, increasing cytotoxic activity, and downregulating inhibitory receptors including TIM-3, NKG2A, and TIGIT (Gong et al., 2025). This immunostimulatory effect appears to be mediated, at least in part, by lipoteichoic acid within SA-EVs through activation of the Toll-like receptor 2 (TLR2)-MyD88-NF-κB signaling pathway. Consistent with these *in vitro* observations, oral administration of SA-EVs in MRL/lpr mice exacerbates systemic inflammation and lupus nephritis, as reflected by enhanced renal NK cell activation, increased immune complex deposition, greater inflammatory cell infiltration, and more severe renal pathology. Moreover, NK cell depletion significantly prolonged the survival time of SA-EV-treated animals. Overall, these findings expand the concept of SA-EV as a virulence related factor, indicating that they can not only mediate local epithelial damage, but also systemic immune dysregulation, thereby contributing to the formation of the pathogenic spectrum of *S. anginosus*.

## 4 Clinical infections and pathogenic mechanisms of *S. anginosus*

### 4.1 Infections of *S. anginosus*

*S. anginosus* is frequently implicated in localized pyogenic infections arising from mucosal and oral sites. It is commonly isolated from dental abscesses, periodontal infections, and oral mucosal lesions, highlighting its clinical relevance in odontogenic and adjacent head-and-neck disease (Robayo et al., 2019). Under this contiguous anatomical context, *S. anginosus* has also been associated with peritonsillar abscess, Ludwig’s angina, sinusitis, and otitis media (Kaplan et al., 2022).

Beyond the oral cavity and upper aerodigestive tract, *S. anginosus* is well-documented in deep-seated thoracic and abdominal infections with a suppurative character. Reported manifestations include pneumonia, lung abscess (Hu et al., 2020), and empyema (Reis-Melo et al., 2020), as well as intra-abdominal infections such as hepatic abscess (Ganju et al., 2017) and peritonitis (Terzi et al., 2016). Collectively, these presentations underscore the prominent association of *S. anginosus* with deep pyogenic disease.

In addition to localized and deep tissue infections, *S. anginosus* can also cause invasive disease, particularly in vulnerable hosts. Bacteremia (Matsuda et al., 2020) and sepsis have been reported in association with this organism (Masood et al., 2016) and may be accompanied by considerable clinical severity. *S. anginosus* has likewise been identified as a cause of infective endocarditis (Forster et al., 2013; Rose et al., 2017; Finn et al., 2018; Dadeboyina et al., 2020), further extending its clinical significance beyond localized suppurative infection.

A broader extraoral spectrum has also been described for *S. anginosus*, including skin and soft tissue infections and musculoskeletal infections such as osteomyelitis. In one recent cohort study, skin and soft tissue infections accounted for a substantial proportion of clinical isolates (Kaplan et al., 2022), indicating that these manifestations are not uncommon in routine clinical settings. Notably, more severe or extensive infections appear to be reported more frequently in immunocompromised individuals and in patients with underlying conditions such as cirrhosis and diabetes mellitus (Ribeiro et al., 2020; Kaplan et al., 2022), emphasizing the opportunistic nature of this organism.

In addition to its established role in pyogenic and invasive infections, emerging evidence has linked intestinal enrichment or colonization by *S. anginosus* to stroke and adverse cardiovascular outcomes (Tonomura et al., 2025). However, heste observations should currently be interpreted as associative rather than causal, and their biological and clinical significance remains to be clarified. Overall, the clinical spectrum of *S. anginosus* is characterized by suppurative, deep-seated, and opportunistic features, providing an important clinical framework for subsequent discussion of its virulence traits and pathogenic mechanisms.

### 4.2 Infectious pathogenic mechanisms of *S. anginosus*

**FIGURE 2:**
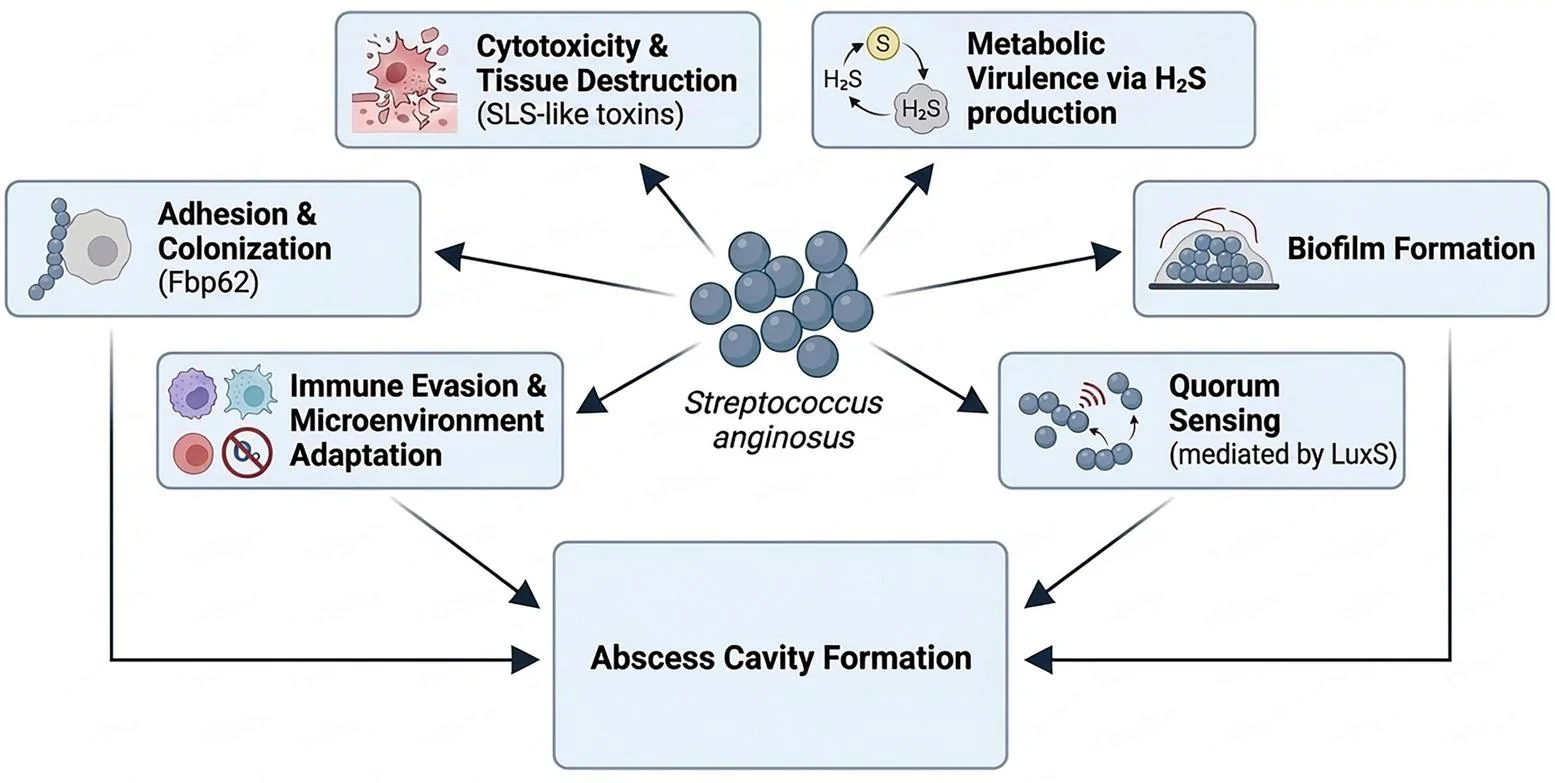
Schematic overview of the virulence traits of *Streptococcus anginosus* involved in abscess formation. The pathogenicity of *S. anginosus* is associated with adhesion and colonization, cytotoxicity and tissue destruction, H_2_S-associated metabolic virulence, biofilm formation, quorum sensing, and immune evasion/microenvironmental adaptation, which together contribute to abscess cavity formation.

The virulence determinants of *S. anginosus* appear to act synergistically in driving localized pyogenic infection. FBP62 has been implicated in bacterial adhesion and colonization of host tissues (Kodama et al., 2018), while SLS is associated with direct cellular injury and tissue necrosis (Tabata et al., 2019). In addition, H_2_S may further amplify local inflammation, thereby contributing to tissue damage (Takahashi et al., 2011). Biofilm formation has been implicated in persistent colonization, antibiotic tolerance, and the establishment of a protective local microenvironment (Petersen et al., 2006), while the LuxS/Auto-inducer 2 (AI-2) quorum-sensing system has been associated with biofilm regulation and reduced antibiotic susceptibility (Ahmed et al., 2007). Together, these properties contribute to immune evasion and chronic persistence at infected sites. In line with these observations, *S. anginosus* is capable of driving purulent inflammation, tissue destruction, and the formation of abscesses or cystic lesions (Figure 2).

Persistent inflammation arising from such suppurative processes may disrupt local tissue homeostasis and prolong inflammatory stimulation. However, the extent to which these localized pathogenic features can be directly extrapolated to gastric epithelial dysplasia or gastric carcinoma remains uncertain. At present, pyogenic infection is more appropriately viewed as evidence of the organism’s inflammatory and tissue-injuring potential rather than as direct proof of a linear transition from localized infection to malignant transformation.

## 5 *S. anginosus* and gastric diseases

### 5.1 Oral-gastric axis colonization

The human oral microbiome is a highly complex ecosystem, predominantly composed of bacteria, with a richness and diversity second only to that of the gut. Several studies have demonstrated that a substantial proportion of gastric microbiota associated with gastric diseases originate from the oral cavity (Xia et al., 2025). *S. anginosus* is a common oral commensal, which is more frequently detected in oral inflammatory conditions such as gingivitis and dental abscesses (Terzi et al., 2016). Meanwhile, it has also been found enriched in the gastric mucosa of patients with gastric cancer (Sasaki et al., 1998).

Current evidence for oral-gastric dissemination of *S. anginosus* is suggestive rather than definitive. Population-based metagenomic studies using matched saliva-stool datasets have provided strain-level evidence for oral-gut transmission of *Streptococcus* species, supporting the broader possibility that oral-derived *Streptococci* may disseminate along the gastrointestinal tract (Qin et al., 2026). But direct evidence for stepwise oral-to-gastric translocation of *S. anginosus* in humans remains limited. Likewise, the enrichment of *S. anginosus* in gastric cancer-associated microbial communities suggests a role in gastric microbial remodeling; however, it remains unclear whether the bacterium acts as a driver, a passenger, or merely a context-dependent co-enriched organism. Experimental studies further showed that exposure to *S. anginosus* induces acute gastritis and progressive gastric histopathological changes in conventional and germ-free murine models, supporting its pathogenic potential in the gastric environment (Fu et al., 2024). However, these findings should be viewed as evidence of biological plausibility, rather than as proof of a fixed transmission route, stable human gastric colonization, or a direct causal role in gastric carcinogenesis. Consistent with oral-gastric link, oral microbial dysbiosis has also been proposed as a potential non-invasive indicator of gastric cancer risk, within which *S. anginosus* may represent one signature component (Sun et al., 2018).

Of note, most mechanistic studies linking *S. anginosus* to gastric carcinogenesis have been derived from Chinese cohorts. Nevertheless, clinical isolates of *S. anginosus* have also been recovered from gastric biopsy specimens of gastric cancer-risk patients in South America, and whole-genome sequences of such gastric-origin isolates are now publicly available for cross-population comparative analysis (Mannion et al., 2026).

### 5.2 Potential mechanisms of *S. anginosus* in gastric cancer

**FIGURE 3:**
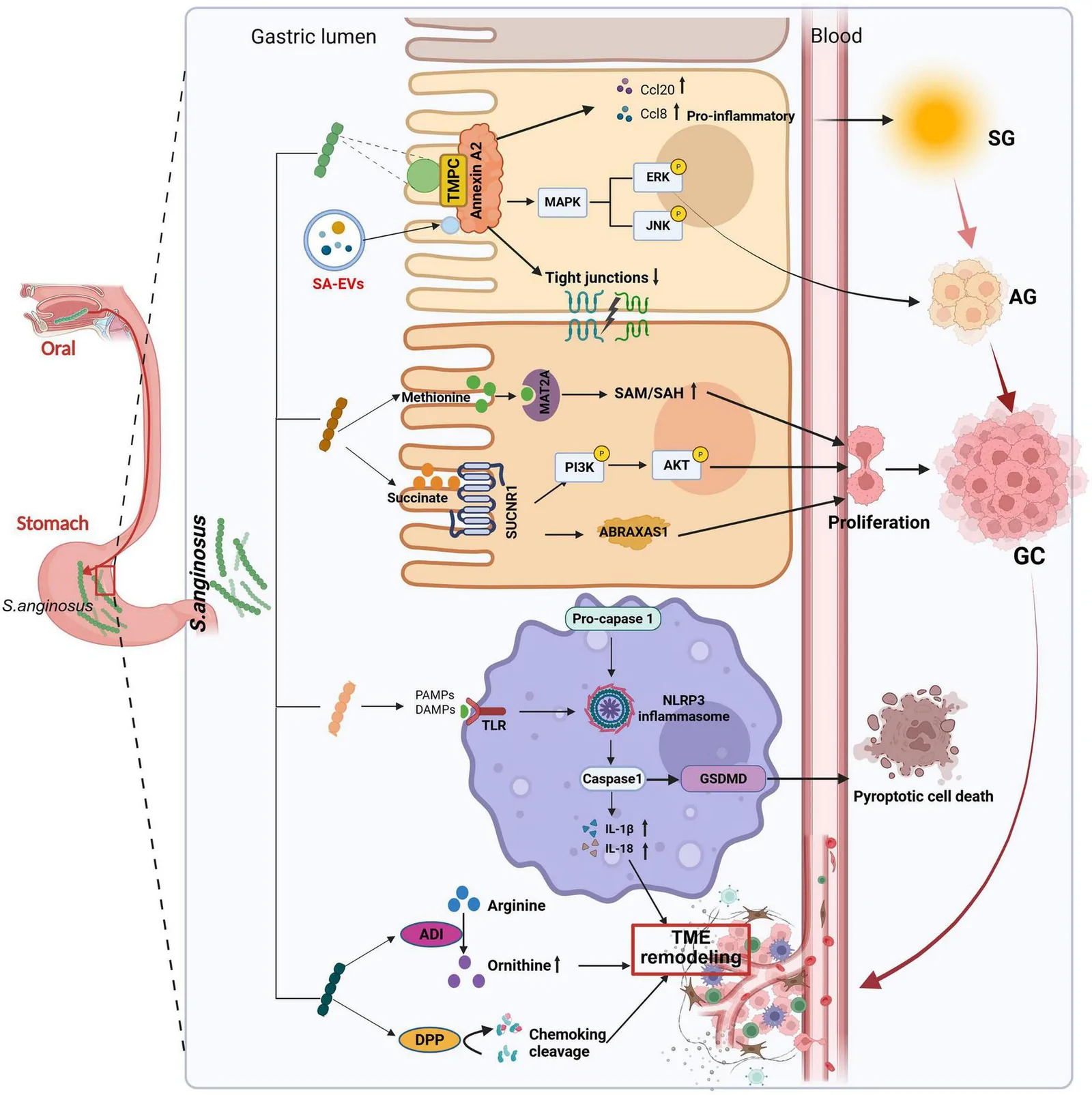
Schematic representation of the proposed mechanisms by which *Streptococcus anginosus* contributes to gastric cancer carcinogenesis. *S. anginosus* may promote gastric carcinogenesis through multiple pathways, including TMPC-ANXA2-MAPK signaling, GSDMD/caspase-1/NLRP3-mediated pyroptosis, succinate-SUCNR1-ABRAXAS1-PI3K/AKT signaling, and methionine metabolism-associated SAM synthesis. These pathways collectively enhance cell proliferation, inflammatory cell death, and malignant progression.

Current evidence suggests that the potential contribution of *S. anginosus* to gastric carcinogenesis may involve multiple, non-mutually exclusive mechanisms, including epithelial injury and dysregulation, immune and tumor microenvironment remodeling, pyroptosis-associated inflammatory amplification, metabolic crosstalk with the host, and context-dependent interaction with *Helicobacter pylori* (*H. pylori*, HP) (Figure 3).

#### 5.2.1 Gastric mucosal injury and epithelial dysregulation

*S. anginosus* has been detected at increased abundance in the gastric mucosa of patients with gastric cancer, suggesting an association with gastric pathology. Experimental studies further showed that, in conventional and germ-free murine models, *S. anginosus* can persist in the gastric mucosa and is associated with acute gastritis and progressive histopathological changes, including chronic gastritis, parietal cell atrophy, mucinous metaplasia, and gastric epithelial dysplasia. In carcinogen-treated models, this organism has also been reported to enhance gastric tumorigenesis and promote the growth of gastric cancer xenografts under experimental conditions (Fu et al., 2024). Mechanistic studies suggest that these effects may involve direct epithelial interactions. *S. anginosus* colonization was associated with impaired gastric barrier function, increased epithelial proliferation, reduced apoptosis. These changes were linked to the bacterial surface protein TMPC, which directly interacts with ANXA2 on gastric epithelial cells. The TMPC-ANXA2 interaction has been reported to facilitate bacterial attachment and colonization. It also engages oncogenic signaling pathways, including Ras/MAPK and phosphatidylinositol 3-kinase (PI3K)-AKT signaling. Specifically, this interaction activates ERK1/2, JNK, and AKT, and induces downstream effectors such as c-Jun, Cyclin D1, and c-Myc (Fu et al., 2024). These signaling changes may help explain the enhanced epithelial proliferation and tumor-promoting phenotypes observed under experimental conditions.

#### 5.2.2 Immune and tumor microenvironment remodeling

*S. anginosus* was considered associated with gastric cancer progression through modulation of the tumor immune microenvironment. Multi-omics analyses have identified a molecular subtype characterized by high *S. anginosus* abundance and poorer prognosis, suggesting a link between this organism and tumor-associated immune or metabolic states. In cell-based mechanistic analyses, *S. anginosus* has been reported to promote gastric cancer cell proliferation and metastatic behavior with reduced CD8^+^ cytotoxic T-cell differentiation and infiltration. One proposed mechanism involves altered arginine metabolism with increased ornithine production, which may contribute to immune remodeling and attenuated anti-tumor responses (Yuan et al., 2024). In addition, *S. anginosus* secretes an Xaa-Pro dipeptidyl peptidase (DPP) that is capable of cleaving chemokine-related substrates, providing a potential mechanism for local immune dysregulation and tumor microenvironment remodeling (Suzuki et al., 2026). Although these observations are mechanistically informative, they currently support a candidate immunomodulatory role rather than a definitive and universally established mechanism in human gastric cancer.

#### 5.2.3 Pyroptosis-associated inflammatory amplification

A preprint study demonstrated that *S. anginosus* supernatant triggers pyroptosis in M0 macrophages via NLRP3-caspase-1-Gasdermin D (GSDMD) axis, leading to robust secretion of pro-inflammatory IL-1β and IL-18. Conditioned medium from *S. anginosus*-challenged macrophages promoted proliferation, migration and epithelial-mesenchymal transition (EMT) in gastric cancer cells, and induced intestinal metaplasia-associated markers, Caudal-related homeobox transcription factor 2 (CDX2), MUC2, and Krüppel-like factor 4 (KLF4) in gastric epithelial GES-1 cells. And all these oncogenic phenotypes were abrogated by the NLRP3 inhibitor MCC950. Untargeted metabolomics further identified 15-Hydroperoxy-5,8,11,13-eicosatetraenoic Acid [15(S)-HPETE] as a key lipid mediator that directly drives malignant transformation and metaplastic changes. Of note, these findings are derived from *in vitro* experiments with limited clinical sample size; as an un-peer-reviewed preprint, independent *in vivo* validation is still required (Han et al., 2026).

#### 5.2.4 Metabolic crosstalk with the host

Metabolic crosstalk has emerged as another candidate mechanism. Recent studies have shown that the methionine biosynthesis pathway was enriched in gastric cancer patients with higher *S. anginosus* abundance. As the methionine derived from *S. anginosus* has been reported to exert tumor-promoting effects in gastric cancer cells and murine models. In this context, bacterial-derived methionine may enter the host methionine cycle and be converted by methionine adenosyltransferase 2A (MAT2A) into S-adenosylmethionine (SAM), which is a major cellular methyl donor that supports DNA, RNA, and histone methylation. Thereby it contributes to epigenetic regulation and other methylation-associated processes. Consistently, gastric cancer tissues with high *S. anginosus* abundance exhibited increased levels of SAM and S-adenosyl-L-homocysteine (SAH), and methionine treatment elevated SAM/SAH levels in gastric cancer cell lines, supporting enhanced methionine-cycle flux (Zhou et al., 2026). In parallel, the *metE* gene, which is involved in methionine biosynthesis, was prevalent in gastric cancer-associated samples, and *metE* deletion attenuated methionine production and reduced the tumor-promoting phenotype in experimental settings. Together, these findings suggest that bacterial-derived methionine may act as a mediator of host-microbe metabolic interaction in gastric cancer, potentially at least in part through promoting SAM production and downstream methylation-related reprogramming.

Similarly, succinate has been implicated as another metabolite potentially involved in *S. anginosus*-associated gastric cancer progression. Experimental evidence suggests that *S. anginosus*-derived succinate can engage succinate receptor 1 (SUCNR1) on gastric epithelial cells and activate downstream ABRAXAS1/PI3K/AKT signaling, thereby promoting proliferative and invasive phenotypes in gastric cancer cells (Liao et al., 2026). While these studies provide mechanistic support for a metabolic contribution, they do not yet establish whether these metabolites are dominant drivers in human gastric carcinogenesis or how these pathways interact with broader microbial and host metabolic networks.

Complementary *in vitro* work further contrasted the oncogenic mechanisms of *H. pylori* and *S. anginosus* in gastric epithelial cells: while *H. pylori* exerted pro-tumorigenic effects mainly via direct cell-contact-dependent pathways, *S. anginosus* acted predominantly through metabolite-driven signaling, highlighting the importance of secreted bacterial products for *S. anginosus*-associated gastric pathology (Luan et al., 2026).

#### 5.2.5 Context-dependent interaction with *H. pylori*

Another factor to consider is the potential interaction between *S. anginosus* and *H. pylori*, which is a major microbial risk factor for gastric cancer. Clinical observations showed a moderate positive correlation between *S. anginosus* and *H. pylori* abundance in gastric carcinoma tissues, whereas such an association was not observed in gastritis tissues. In non-neoplastic gastric mucosa from patients with chronic gastritis, *S. anginosus* abundance was decreased under *H. pylori* infection, whereas in gastric cancer tissues its abundance increased in parallel with *H. pylori*, suggesting that the relationship between these organisms may be disease-stage dependent (Zhou et al., 2022).

Co-infection studies further indicate that *S. anginosus* and *H. pylori* may cooperatively enhance gastric inflammation compared with mono-infection in murine models (Fu et al., 2024). Clinical dataset analyses also suggest that co-infected individuals may have a higher risk of atrophic gastritis, intestinal metaplasia, and gastric cancer than those infected with *H. pylori* alone. The current data supports the possibility of context-dependent cooperation between *S. anginosus* and *H. pylori*, but have not establish a uniform synergistic mechanism across all stages of gastric disease.

## 6 Clinical management and translational application

### 6.1 Diagnostic strategies for *S. anginosus*

At present, no gold standard is available for the identification of *S. anginosus*. Cultivation methods and the available biochemical tests are insufficient for precise species-level identification (Wenzler et al., 2015; Kragha, 2016). MALDI-TOF MS can reliably identify *S. anginosus* and *S. constellatus* at the species level, but it does not reliably identify *S. intermedius* (Woods et al., 2014). Molecular approaches have expanded the toolkit for detecting and characterizing *S. anginosus* across both clinical infection and microbiome-associated settings. PCR restriction fragment length polymorphism (PCR-RFLP) has also been used as a confirmatory method for identifying members of the *S. anginosus* group and for differentiating *S. anginosus* from *S. constellatus* (Liu et al., 2006). Real-time PCR coupled with melting curve analysis enables relatively rapid identification of members of the *S. anginosus* group and can further distinguish *S. anginosus* subgroups associated with clinical ribotypes (Desar et al., 2008). For diagnostically challenging or culture-negative invasive infections, cell-free DNA-based assays may provide a useful adjunct for pathogen detection (Da Costa et al., 2026), although their performance and clinical utility in *S. anginosus*-specific settings still require broader validation.

In parallel, 16S rRNA gene sequencing combined with qPCR has been used primarily to assess relative abundance and differential microbial profiles in non-invasive samples, including feces, rather than to establish disease causality. On this basis, the combined detection of *S. anginosus* and *S. constellatus* has been proposed as a candidate non-invasive biomarker strategy for gastric cancer risk stratification or early detection (Zhou et al., 2022). However, such findings currently support association and exploratory biomarker potential and should be interpreted cautiously pending validation in larger, prospective, and externally replicated cohorts.

More recently, a PCR-CRISPR/Cas12a platform targeting *S. anginosus* 16S rDNA has been developed, with reported high analytical sensitivity and specificity in fecal samples (Zheng et al., 2025). This approach may be valuable for sensitive organism detection in research and potentially in future clinical workflows, but its current evidence base remains largely methodological. Overall, advances in molecular diagnostics are improving the detection of *S. anginosus* and may facilitate studies of its relationship to gastric mucosal pathology; nevertheless, the transition from microbial detection to clinically actionable screening will require rigorous standardization, prospective validation, and careful control for confounding factors (Table 2).

**TABLE 2:** Diagnostic approaches for the detection and characterization of *S. anginosus*.

Method	Main purpose	Sample type	Advantages	References
MALDI-TOF MS	Species-level identification of *S. anginosus* and *S. constellatus*	Cultured clinical isolates	Rapid and reliable species-level identification for *S. anginosus* and *S. constellatus*	Woods et al., 2014
PCR restriction fragment length polymorphism (PCR-RFLP)	Confirmatory identification of *S. anginosus* and differentiation from *S. constellatus*	Clinical isolates/bacterial DNA	Useful as a confirmatory method for the identification of *S. anginosus* and its differentiation from *S. constellatus*	Liu et al., 2006
Real-time PCR with melting curve analysis	Identification of members of the *S. anginosus* group (SAG) and discrimination of *S. anginosus* subgroups associated with clinical ribotypes	Clinical infection samples	Relatively rapid; provides subgroup discrimination	Desar et al., 2008
Cell-free DNA-based assays	Adjunctive pathogen detection in diagnostically challenging or culture-negative invasive infections	Blood or other body fluids containing cf DNA	Potentially useful when conventional culture is negative	Da Costa et al., 2026
16S rRNA gene sequencing combined with qPCR	Assessment of relative abundance and differential microbial profiles	Non-invasive samples, including feces	Useful for microbiome association studies; non-invasive	Zhou et al., 2022
PCR-CRISPR/Cas12a	Sensitive and specific detection of *S. anginosus*	Fecal samples	Reported high analytical sensitivity and specificity	Zheng et al., 2025

Although *S. anginosus* is often considered susceptible to several commonly used agents, accumulating evidence indicates that this species can harbor diverse antimicrobial resistance and tolerance determinants. Reported resistance-associated genes include erm(B), tet(M), lnu(C), and aminoglycoside resistance genes (Clermont and Horaud, 1990; Gravey et al., 2013), supporting reduced susceptibility to macrolides, tetracyclines, and lincosamides in at least a subset of strains. In addition, modulation of antibiotic susceptibility by the LuxS/AI-2 quorum-sensing system (Ahmed et al., 2007) and vancomycin tolerance in clinical isolates (Kwack et al., 2022) suggest that both genetic and physiological factors may contribute to treatment difficulty. At the same time, earlier data indicate that many strains remain susceptible to cefotaxime and glycopeptides (Jacobs and Stobberingh, 1996), underscoring the heterogeneity of resistance phenotypes across isolates.

A recurring theme in recent work is the importance of mobile genetic elements, particularly integrative and conjugative elements (ICEs), in shaping the resistome of *S. anginosus*. Several studies have identified ICEs carrying resistance genes such as *erm(B)* and tetracycline-associated determinants, and experimental conjugation data suggest that some of these elements can move across streptococcal species and even into enterococci (Yi et al., 2024; Wang et al., 2025a, b). Mobilome analyses further indicate that *S. anginosus* may serve as a reservoir of antibiotic resistance genes (ARGs)-associated ICEs, prophage-related elements, and composite mobile structures, highlighting its potential role in interspecies dissemination of resistance within polymicrobial communities (Wang et al., 2025a).

From a clinical perspective, these findings are relevant because they may contribute to persistence, recurrence, and therapeutic complexity in *S. anginosus* infection. However, current evidence is derived largely from genomic analyses, small isolate collections, and experimental transfer systems, and the frequency with which these mobile elements influence treatment outcomes in human disease remains insufficiently defined. Thus, antimicrobial resistance in *S. anginosus* should be viewed as an emerging and heterogeneous challenge rather than a uniformly established phenotype, warranting continued surveillance and closer integration of genomic and clinical susceptibility data.

### 6.2 Antimicrobial therapy and procedural management

Antimicrobial therapy remains the cornerstone of clinical management for *S. anginosus* infections, while drainage or surgical intervention is often required in severe, abscess-forming, or otherwise complicated cases. In routine practice, treatment selection should be guided by the infection site, disease severity, host status, and, where available, isolate-specific susceptibility profiles. Fluoroquinolones, including levofloxacin, have been described as salvage options in selected refractory cases, such as infections not responding to first-line therapy or situations in which surgical management is not feasible (Inami et al., 2025). However, such observations should be interpreted in the context of limited clinical evidence and should not be generalized as a standard approach across all *S. anginosus* infections.

At the same time, reliance on empirical or prolonged antibiotic therapy has important limitations. Broad-spectrum exposure may disrupt the resident microbiota (Knoop et al., 2016), and inadequate adherence, inappropriate prescribing, and nonstandard regimens can promote persistence, recurrence, and resistance selection (Dascãlu et al., 2023). Biofilm formation and adaptive bacterial responses may further reduce treatment effectiveness in some settings (Tavernier et al., 2018). Taken together, these issues support the need for more precise antimicrobial stewardship, susceptibility-guided treatment, and better-defined management algorithms, particularly for recurrent, invasive, or difficult-to-eradicate infections.

### 6.3 Probiotics as adjunctive microbiota-modulating strategies

Probiotics have attracted interest as a possible adjunctive strategy for modulating host-microbe interactions relevant to *S. anginosus* colonization and infection. In general, probiotics can influence epithelial barrier integrity, immune tone, and microbial competition within the gastrointestinal tract (Suez et al., 2019; Dey, 2025). Commonly used genera include *Lactobacillus*, *Bifidobacterium*, and *Saccharomyces* (Hill et al., 2014), although their effects are highly strain-specific rather than genus-specific (Hill et al., 2014; Yang et al., 2024). In principle, such organisms may reduce colonization burden or alter ecological conditions through mucin modulation, antimicrobial metabolite production, and competitive exclusion of pathogens.

However, direct evidence supporting probiotic-based control of *S. anginosus*, particularly in the context of gastric colonization, gastric precancerous lesions, or tumor-associated microbial states, remains limited. Much of the available rationale is extrapolated from the broader probiotic literature or from *in vitro* observations, including studies showing that *Bifidobacterium* species can inhibit pathogenic bacteria through organic acid production (Fukuda et al., 2011), antimicrobial peptide production and inhibition of adhesion/invasion (Moroni et al., 2006), quorum-sensing interference (Kim et al., 2012), and immune modulation (Fanning et al., 2012). Accordingly, probiotics may represent a candidate adjunct for microbiota modulation and barrier support, but their organism-specific efficacy, optimal strain selection, and clinical relevance in *S. anginosus*-associated disease require dedicated validation in well-designed experimental and clinical studies.

### 6.4 Prevention and vaccine development

Given the clinical burden of invasive and recurrent *S. anginosus* infection, preventive strategies, including vaccine development, are of growing interest. At present, however, this field remains largely preclinical, and no licensed vaccine is available. Recent studies using reverse vaccinology and immunoinformatics have proposed multi-epitope subunit vaccine candidates with favorable predicted antigenicity and molecular interaction profiles *in silico* (Xu et al., 2025). These findings are useful for antigen prioritization, but they should be interpreted as computationally derived hypotheses rather than evidence of established protective efficacy.

A similar level of caution applies to TMPC-targeted vaccine designs motivated by the proposed role of the TMPC-ANXA2-MAPK axis in epithelial interaction and gastric pathology. Multi-epitope and mRNA-based TMPC-centered constructs have shown encouraging performance in immune simulation and docking analyses, including predicted interactions with immune receptors and induction of modeled humoral or cellular responses (Zhu et al., 2026). Nevertheless, these data remain preclinical and largely *in silico*, and key questions regarding *in vivo* immunogenicity, protective efficacy, strain coverage, safety, and disease-context specificity remain unresolved. Therefore, vaccine development against *S. anginosus* should currently be viewed as a promising but early-stage direction, with translational relevance contingent on rigorous animal and, eventually, clinical validation.

## 7 Conclusion and future perspectives

Despite growing interest in the pathogenic and clinical significance of *S. anginosus*, several important knowledge gaps continue to limit mechanistic interpretation and translational application. Current evidences support that *S. anginosus* is more than a bystander in some inflammatory and gastric disease settings, but the strength of evidence varies substantially across human association studies, experimental models, and emerging mechanistic analyses.

Several priorities may guide our future work. First, large-scale longitudinal cohorts are needed to clarify whether *S. anginosus* colonization is temporally associated with progression from gastric precancerous lesions to gastric cancer and whether intervention against this organism alters disease trajectories. Furthermore, most mechanistic investigations into the gastric oncogenic role of *S. anginosus* have been performed using East Asian patient materials. Although gastric-derived *S. anginosus* strains have been isolated from geographically distinct populations, cross-regional functional validation is urgently needed to assess whether current findings can be generalized globally. Second, the oral-gastrointestinal axis of *S. anginosus* requires deeper investigation, particularly with respect to translocation routes, niche adaptation, and interactions with resident microbial communities. Third, *S. anginosus*-derived extracellular vesicles represent an additional candidate mechanism that warrants further study in relation to cargo composition, host targeting, mucosal immunity, and metabolic regulation.

From a translational perspective, future efforts should also focus on validating rapid and noninvasive diagnostic platforms, developing better-defined therapeutic strategies that balance antimicrobial efficacy with microbiota preservation, and continuing surveillance of antimicrobial resistance. Candidate preventive approaches, including vaccines targeting proteins such as TMPC and FBP62, remain of interest but require substantial preclinical and clinical validation. Overall, *S. anginosus* may represent a candidate microbial biomarker and potential intervention target in gastric disease; however, its strain specificity, causal relevance, and clinical utility remain to be established in well-controlled studies.
